# Defining Transcriptional Regulatory Mechanisms for Primary let-7 miRNAs

**DOI:** 10.1371/journal.pone.0169237

**Published:** 2017-01-04

**Authors:** Xavier Gaeta, Luat Le, Ying Lin, Yuan Xie, William E. Lowry

**Affiliations:** 1 Broad Stem Cell Center, University of California Los Angeles, Los Angeles, California, United States of America; 2 Molecular Biology Institute, University of California Los Angeles, Los Angeles, California, United States of America; 3 Molecular, Cell and Developmental Biology, University of California Los Angeles, Los Angeles, California, United States of America; 4 Jonsson Comprehensive Cancer Center, University of California Los Angeles, Los Angeles, California, United States of America; Kunming University of Science and Technology, CHINA

## Abstract

The *let-7* family of miRNAs have been shown to control developmental timing in organisms from *C*. *elegans* to humans; their function in several essential cell processes throughout development is also well conserved. Numerous studies have defined several steps of post-transcriptional regulation of *let-7* production; from pri-miRNA through pre-miRNA, to the mature miRNA that targets endogenous mRNAs for degradation or translational inhibition. Less-well defined are modes of transcriptional regulation of the pri-miRNAs for *let-7*. *let-7* pri-miRNAs are expressed in polycistronic fashion, in long transcripts newly annotated based on chromatin-associated RNA-sequencing. Upon differentiation, we found that some *let-7* pri-miRNAs are regulated at the transcriptional level, while others appear to be constitutively transcribed. Using the Epigenetic Roadmap database, we further annotated regulatory elements of each polycistron identified putative promoters and enhancers. Probing these regulatory elements for transcription factor binding sites identified factors that regulate transcription of *let-7* in both promoter and enhancer regions, and identified novel regulatory mechanisms for this important class of miRNAs.

## Introduction

The *let-7* family of miRNAs were first identified in *C*. *elegans* as a single heterochronic factor controlling developmental timing[1, 2]. Since then, this family of miRNAs has been shown to play somewhat equivalent roles in all bilaterian organisms, and the *let-7*s were the first miRNAs identified in humans[1, 3]. The *let-7*s have now been implicated in differentiation and maturation of many tissues during development *in vivo* and *in vitro*[4–7]^3–8^. As with other miRNAs, the initial pri-*let-7* transcripts are first transcribed by RNA polymerase II, then processed via the canonical pathway through the pre-miRNA stage generated by the action of Drosha/DGCR8. The pre-miRNA is then processed in the cytoplasm by Dicer to generate the mature version of the miRNA[8–10]. In addition, in the case of *let-7* miRNAs, other processes such as uridylation are used to stabilize or destabilize miRNAs[11–13]. LIN28A and LIN28B are RNA binding proteins that regulate several of these processing steps to control levels of mature *let-7* transcripts[14, 15]. Over evolution, *let-7* isoforms have expanded such that the human genome contains 9 isoforms. The study of regulation of the *let-7* family of miRNAs has focused on these processing steps, but less is understood about how the pri-*let-7* transcripts are regulated by transcription prior to any processing.

Studies in *C*. *elegans*, where the activity and expression of *let-7* is regionally and temporally constrained, have attempted to clarify transcriptional regulation from the single *let-7* locus. Two regulatory regions upstream of the locus were identified as the temporally regulated expression binding site (TREB) and the *let-7* transcription element (LTE), and many studies have tested the binding and transcriptional control exerted by several TFs including elt-1 and daf-12[2, 16–18]. These sequences are not present upstream of mammalian *let-7* gene, and there are not similarly consistently present sequences near all the different *let-7* loci. In higher organisms, a different system for regulating *let-7* miRNA transcription must have been established.

The study of mammalian pri-*let-7* transcription is hampered by the relative scarcity of the transcript which is processed immediately in the nucleus and therefore difficult to detect. We previously took advantage of a method that allows for the capture of nascent RNA transcripts, which are still associated with the chromatin from which they are transcribed, to carefully annotate pri-*let-7* transcripts[19, 20]. Another group later induced *pri-let-*7 accumulation in the context of DGCR8 knockout, and validated with RACE PCR that primary *let-*7 transcripts have multiple isoforms, some of which aligned nearly identically to our observed annotation patterns and varied in different cellular contexts[21]. From these annotations, it is clear that many *let-7* family members are transcribed within very long (up to 200KB), often polycistronic transcripts[20, 21]. While some studies have identified transcriptional models of *pri-miRNAs* in higher organisms, the lack of proper annotation left the precise regulatory motifs for human *let-7* transcripts undefined. Here, after complete annotation of *let-7* transcripts, we attempt to define regulatory motifs for this family of miRNAs by taking advantage of Chromatin-associated RNA-seq and the latest genomic descriptions of chromatin states within *let-7* loci. We model *let-7* transcription in distinct neural paradigms to reveal subsets of *let-7* family members that are transcribed constitutively versus dynamically regulated in particular contexts. Finally, by analyzing publically available data for *let-7* loci, we identify transcription factors that appear to regulate *let-7* transcription by acting at either promoter or enhancer elements enriched in dynamically regulated *let-7* polycistrons.

## Results

### Identification of dynamics of let-7 polycistron transcription

As a first step to determine how *let-7* miRNAs are transcriptionally regulated, we attempted to define developmental models that display dynamism of transcription. We previously identified dynamic transcriptional regulation of some *let-7* family members between neural progenitors that represent distinct developmental stages[20]. This developmental system has been described in our previous studies[19, 20, 22], and has become routine in the field. Initially, human pluripotent stem cells are directed towards a neural fate by changing the media. Then, as neural rosettes are formed, they are manually isolated and expanded in neural proliferation media containing EGF and FGF. To further differentiate towards later lineages, we used growth factor withdrawal, where EGF and FGF are removed and the neural progenitors are forced to differentiate towards neurons and glia. We validated the identity of cells generated at each step by immunostaining for markers typical of each stage of specification (pluripotent: OCT4/NANOG, NPC: SOX2, SOX1, Neuron: MAP2, TuJ1), and consider the culture to be suitable homogenous if the cells are at least 90% positive for combinations of markers.

**Fig 1:**
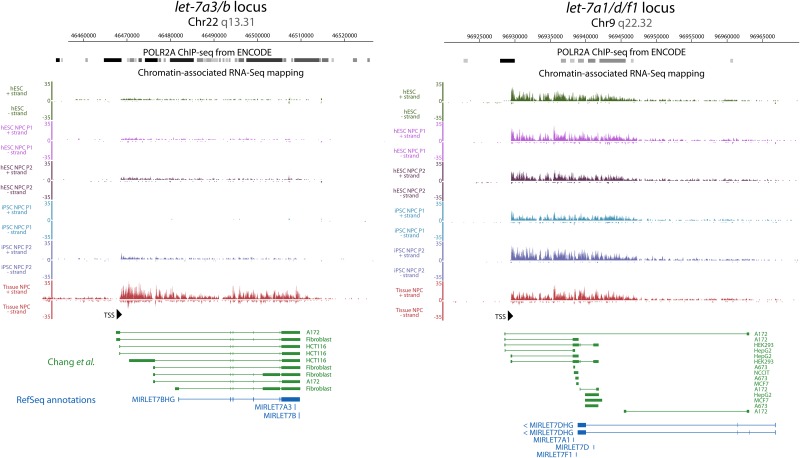
Dynamic transcriptional regulation of some *pri-let-7* transcripts. Chromatin-associated RNA-seq reads were mapped onto two distinct polycistronic *let-7* loci. At left, the *let-7a3/b* locus, is dynamic, while at right, the *let-7a1/d/f1* locus, is constitutively expressed. Reads are shown for ESC, iPSC, PSC-derived NPC, and neural tissue-derived NPC stages. These reads are aligned with validated primary miRNA transcripts from RACE PCR experiments in green and RefSeq annotated genes in blue^25^. Note that Chromatin-associated RNA-seq and RACE PCR annotated transcripts demonstrate the existence of longer transcripts from different transcriptional start sites than suggested by the RefSeq annotation. In the case of *let-7a1/d/f1*, this discrepancy extends to the strand from which initial transcription occurs.

We previously showed that *pri-let-7* transcripts can be identified by Chromatin-associated RNA-seq data[20](NIH GEO Dataset GSE32916). This method captures RNA still associated with chromatin, and therefore represents nascent messages[23]. Our previous analysis initially predicted that some *pri-let-7s* could be dynamically regulated, so we extended these analyses here. Here we show that there is dynamism of *let-7* transcription as measured by Chromatin-associated RNA-seq as witnessed by the fact that the *let-7a3/b* locus is practically silent in pluripotent stem cells, and neural progenitors derived therein, but strongly expressed in tissue derived neural progenitors (Fig 1A). This is consistent with the idea that tissue derived progenitors represent a later stage of development than pluripotent derived progenitors[19, 20].

On the other hand, the same analysis for the *let-7a1/d/f1* locus showed that this polycistron is constitutively expressed across all cell types assayed (Fig 1B). Furthermore, Chromatin-associated RNA-seq also allows for mapping reads which highlighted the fact that *let-7* transcripts are long and sometimes polycistronic. Finally, this allowed for proper annotation of these polycistronic transcripts by actual measurement of message as opposed to the RefSeq annotations that were performed by localizing epigenetic markers. In so doing, we find that the RefSeq annotations underestimate the length of the let-7 polycistrons. The annotations resulting from the Chromatin-associated RNA-seq are then highly overlapping with those in another study, Chang et al[21]. A complete presentation of transcriptional data from the other *let-7* loci as demonstrated by Chromatin-RNA-seq is in (S1 Fig).

**Fig 2:**
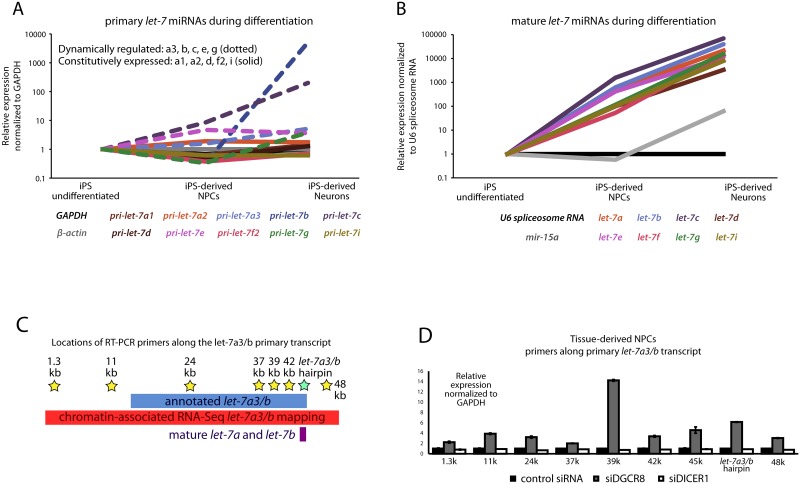
Expression of *pri-let-7* during neural specification. Pluripotent stem cells were differentiated through the neural lineage to neural progenitor cells (NPCs) and then to neurons. Using RT-PCR with primers specific to the *let-7* miRNAs at different stages of processing, we tested changes in expression of the *pri-let-7*s **(A)** and their mature forms **(B)**. While all mature miRNAs increased over the course of differentiation, only a subset (marked with dotted lines), the dynamically regulated *let-7*s, also increased before processing, at the primary *let-7* stage. RT-PCRs were also performed beginning with ES cells. **(C)** Graphic comparing the length of the RefSeq annotated *let-7a3/b* with our predicted transcript. Stars mark primer pairs for RT-PCR along the full transcript. **(D)** RT-PCR of *pri-let-7a3/b* transcript in tissue-derived NPCs, in which transcription is abundant. In control, siDGCR8 (to block Microprocessor function and pri-to-pre conversion), and siDICER (to block pre-to-mature conversion) conditions. When Microprocessor is disabled, the entire *let-7a3/b* transcript accumulates.

Using RNA from cultures previously described [20], we performed Reverse Transcriptase Polymerase Chain Reaction (RT-PCR) to show that as human pluripotent stem cells are specified to neural progenitors, and subsequently into neurons, some primary *let-7* transcripts are strongly induced as measured by RT-PCR (Fig 2). Because pri-miRNAs are transcribed as longer messages, they can be specifically identified by designing at least one of the PCR primers to recognize strictly cDNA made from the portions of the pri-miRNA message not found in pre-mrRNA or mature miRNA. In both developmental scenarios, we observed that a subset of *let-7* family members showed transcriptional induction over developmental time, while other members appeared to be constitutively transcribed (Fig 2A). Using primers that recognize the mature version of miRNA, RNA isolated in a manner that enriches for small RNA, and a specialized cDNA synthesis kit (miScript) we also specifically analyzed levels of mature *let-7s*. We found that the levels of all mature *let-7* family members were strongly induced across development (Fig 2B).

As evidence for the long length of these transcripts, RT-PCR was performed using primers that recognize different regions of the predicted transcript from the *let-7a3/b* locus (Fig 2C). In addition, we posited that this transcript would accumulate in abundance if downstream processing by DGCR8 was inhibited. siRNA-mediated silencing of DGCR8 increased levels of the *let-7a3/b* transcript as measured by all the primers across the entire predicted polycistron. Silencing of DICER, necessary for the final step of miRNA processing, did not change the level of any portion of the *let-7a3/b* transcript (Fig 2D). As further evidence that *let-7* transcripts are polycistronic, the data in Fig 1A and 1B on dynamic versus constitutive indeed showed a shared pattern for those *let-7*s that are in the same polycistron. For instance, the pattern of *let-7a* and *let-7b* was conserved and dynamic in both contexts, while *let-7a1*, *let-7d* and *let-7f1*, which are also polycistronically transcribed, were constitutively expressed in both contexts.

### Identification of potential epigenetic regulation of let-7 polycistrons

**Fig 3:**
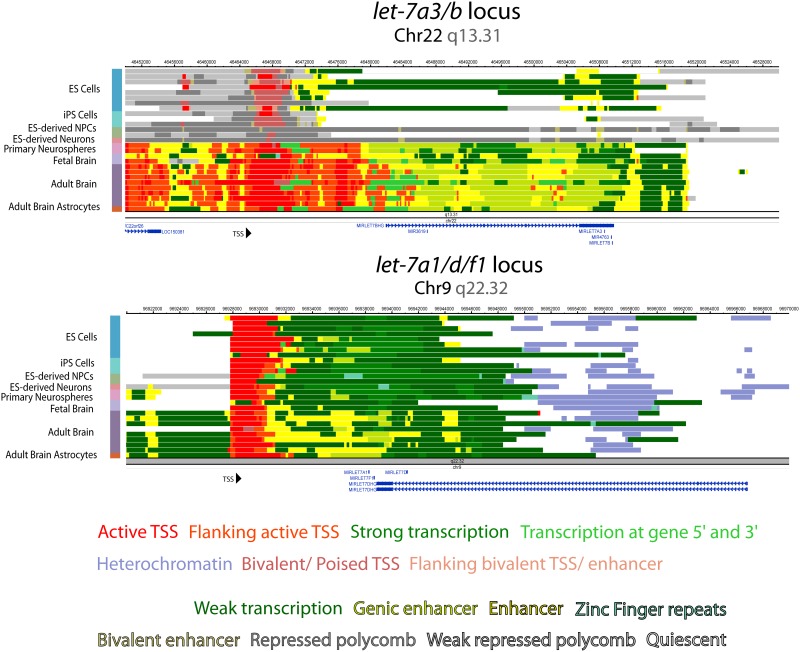
Dynamically and constitutively transcribed *let-7* loci show distinct epigenetic signatures. Computationally imputed chromatin states generated by the ChromHMM algorithm at the same *let-7* loci. Each row represents one biological sample. These states show active transcriptional marks at the predicted TSS for *let-7a1/d/f1* in multiple cell types. At the *let-7a3/b* locus, ES cells, iPS cells, and PSC-derived NPCs have marks consistent with poised promoters, but later in differentiation active TSS marks appear at the same sites, reflecting changes in epigenetic state during neural differentiation. Epigenetic marks in K562 leukemia cells show active transcription at the RefSeq annotated *let-7a3/b* locus.

We then sought to determine whether the dynamic versus constitutive *let-7* polycistrons display distinct regulatory schemes. Using data from the Epigenetic Roadmap, we annotated the chromatin states across each polycistronic *let-7* locus (Fig 3 and S2 Fig). The Roadmap database includes data from dozens of human cell types, including several of the neural lineage and pluripotent stem cells, both highly relevant to our current study[24, 25]. This analysis showed a clear distinction between the regulatory framework for the dynamically regulated *let-7a3/b* locus, versus that of the constitutively expressed *let-7a1/let-7d/let-7f1*locus. The clearest distinction comes in the form of location of epigenetic marks for enhancers and transcriptional start sites (TSSs), where the *let-7a3/b* locus contains several possible start sites and putative enhancers, while the *let-7a1/let-7d/let-7f1* locus appears to only have one predicted start site and regulatory scheme. We posit that having multiple possible TSSs could indicate a message that is dynamically regulated in a variety of settings.

Using these data and the imputed chromatin state model in tamed, we clearly identified TSSs, promoters (active and poised), enhancers, and actively transcribed regions for two of the *let-7* polycistrons (Fig 3). As further evidence for their polycistronic nature, these updated epigenetic data from a wide variety of primary cell types again predicted single, long transcripts across entire loci that encompass multiple *let-7* family members, as opposed to older analyses on transformed cell lines upon which the RefSeq annotations were created. Importantly, some of the genomic state models predicted variation of states in distinct cell types. Notably, the predicted promoter of *let-7a3/b* was shown to be poised in hPSCs and hPSC-derived NPCs, and active and transcribed in later neural derivatives and in brain. This pattern is highly consistent with our own transcriptional data whereby the pri-*let-7a3/b* polycistron was not transcribed significantly until hPSC-derived NPCs were driven further to neurons (Fig 2A and 2B).

### Functionally defining regulators of let-7 transcription

**Fig 4:**
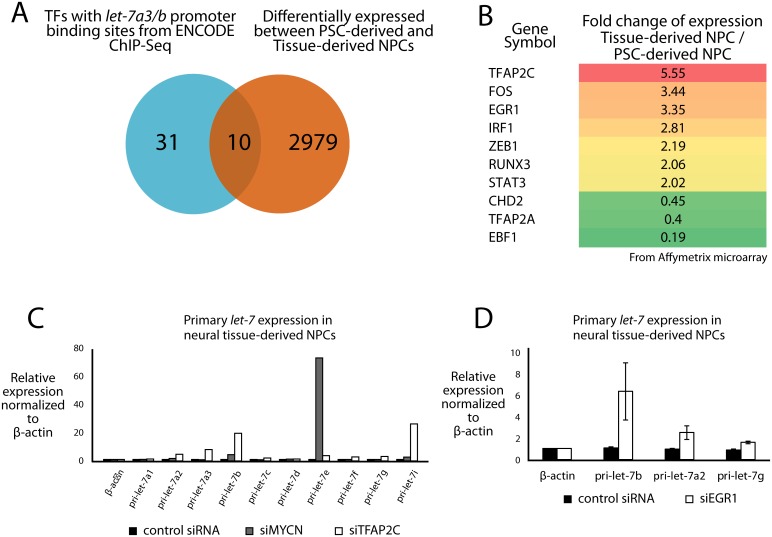
Transcription factors predicted to bind to the *let-7a3/b* promoter regulate primary *let-7a3/b* transcription. **(A)** Comparison of transcription factors with experimentally determined binding sites to the *bona fide let-7a3/b* promoter from the ENCODE database with genes differentially expressed between tissue-derived NPCs (in which *let-7a3/b* is abundantly transcribed) and PSC-derived NPCs (in which it is not). 10 genes were present in both sets, and are shown at right, ranked by their fold change of expression between tissue-derived and PSC-derived NPCs from microarray based gene expression measurements. We knocked down several of these candidate *let-7* regulator transcription factors in tissue-derived NPCs. **(B)** Knockdown of the *TFAP2C* gene encoding the AP-2γ protein, and of the *MYCN* gene increase transcription of several *let-7* genes. Data shown are representative of 3 independent experiments. **(C)** Knockdown of *EGR1* increases transcription of primary *let-7b* and other *let-7* genes. Error bars are ± SEM from n = 3 biological replicates.

Globally, the utility of these analyses was to define more precisely the location of promoters and enhancers for each of the pri-*let-7s*. Taking advantage of the annotation of promoters, we attempted to identify mechanisms of transcriptional regulation of the dynamic versus constitutively regulated *let-7* polycistrons. With a focus on *let-7a3/b*, we searched for transcription factors that could regulate this polycistron through interactions at the promoter. We first used transcription factor ChIP-Seq data from the ENCODE and the Epigenetic Roadmap datasets to detect transcription factor binding sites enriched in this promoter (Fig 4A). We then narrowed the list of candidates to include just those whose expression changes in contexts where *let-7a3/b* transcription also changes. This led to the identification of 10 TFs as defined by previous data showing their ability to both bind DNA and affect transcription of target genes (Fig 4B). To functionally determine whether any of these TFs can affect *let-7a3/b* transcription, we silenced some of them in tissue-NPCs (where transcription of *let-7a3/b* is high) and performed RT-PCR. In addition, we also targeted MYCN as a positive control because of its previously established ability to regulate let-7 transcription [1, 26, 27] and based on its expression pattern in our neurodevelopmental model. Silencing of N-MYC, AP2a, or EGR1 all appeared to lead to an increase in *let-7a3/b* transcription after just two days (Fig 4C and 4D), indicating a role for these TFs in transcriptional regulation of this polycistron.

**Fig 5:**
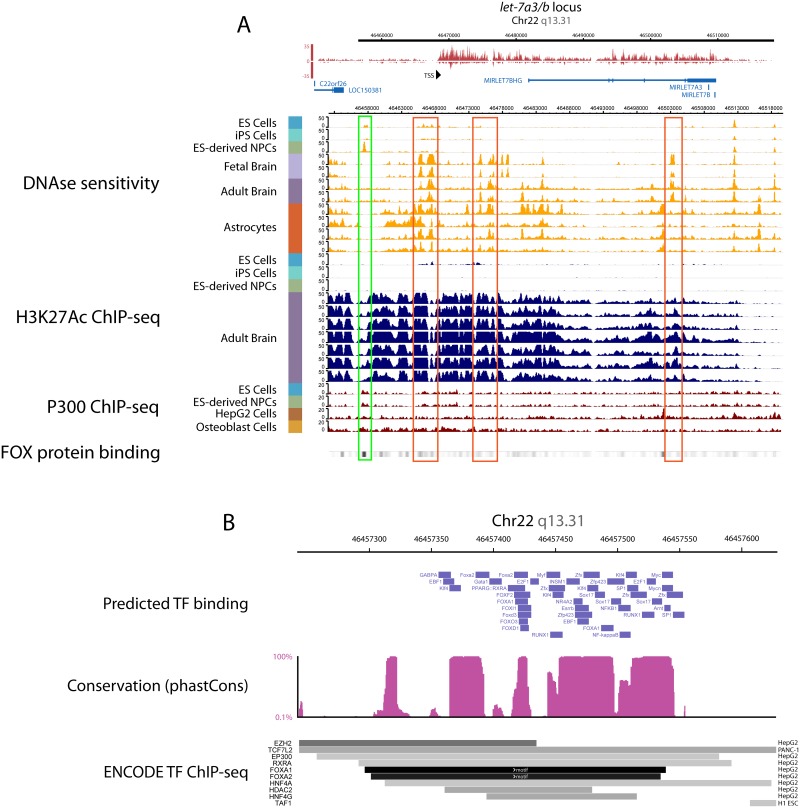
FOX proteins are predicted to bind to putative *let-7a3/b* enhancer regions. **(A)** The predicted existence of an upstream enhancer for the *let-7a3/b* locus was based on the epigenetic state at a region 10kb upstream of the TSS, outlined in green. In addition to being marked by H3K27Ac ChIP-Seq peaks with a localized dip in signal intensity, and peaks for the enhancer-associated histone acetyltransferase protein P300, this region showed dynamic changes in DNAse sensitivity. Note that a large DNAse sensitivity peak appears only in ES-derived NPCs, suggesting a differentiation state-specific chromatin opening at this region. At bottom, relative intensity of forkhead box protein ChIP-Seq from multiple cell types are pooled, with the darkest regions indicating intense FOX protein binding. Outlined in red are similar regions that show DNAse sensitivity beginning at the fetal brain stage that also colocalize with FOX protein binding. **(B)** A zoomed in view of the green region of increased DNAse sensitivity in PSC-derived NPCs. In blue are computationally predicted transcription factor binding sites from the ORCAtk database. The degree of genomic conservation along this region from the PhastCons64 database is shown in purple. At bottom are transcription factor ChIP-seq mapped peaks from the ENCODE database. The regions in green mark forkhead box transcription factor conserved motifs. Note that the forkhead box motifs co-localize with a region of highly conserved sequence, and the redundant binding of the forkhead box motif by many family members predicts that many such proteins can bind there.

To focus on potential regulatory mechanisms at enhancers, we next looked for putative enhancers by looking for regions of enriched DNAse-hypersensitivity, peaks of H3K27ac, and peaks associated with p300 binding in the *let-7a3/b* locus (Fig 5A). Several predicted enhancers, outlined in red rectangles, were highly DNAse sensitive region in Fetal and Adult brain samples but not in PSCs or PSC-derived NPCs. This pattern correlates with the timing of increased *pri-let7a3/b* transcription. A different site, 10kb upstream of the newly annotated TSS, is outlined in a green rectangle, and instead showed DNAse sensitivity only in PSC-derived NPCs, and not in either the undifferentiated or fully differentiated cells in the database. All of these identified regions showed P300 binding, were surrounded by ChIP-seq peaks for acetylated H3K27 in neural samples, and were significant for a specific depletion of histone-associated ChIP-seq binding peaks right at the site of DNAse sensitivity.

We used a similar approach to identify predicted and validated TF binding sites in the enhancer regions as on promoter sequences (Fig 5B). This analysis yielded a strong enrichment of binding by the forkhead box transcription factors (FOX proteins), all of which can bind the same motif: 5'-[AC]A[AT]T[AG]TT[GT][AG][CT]T[CT]-3'[28]. Note the increased intensity of FOX protein ChIP-seq signal within the putative enhancers in Fig 5A. The furthest upstream such region, outlined in green, is shown in more detail, with both predicted and experimental FOX protein binding localizing to one highly conserved area (Fig 5B). The forkhead box TFs contain winged helix domains, which contribute to the pioneer transcription factor activity of the entire family and in some settings could be responsible for observed changes in chromatin accessibility[29–32]. We compared this finding with expression data to subsequently determine which candidate forkhead box TFs could potentially be acting at the *let-7a3/b* enhancer during neural development (Fig 5B).

**Fig 6:**
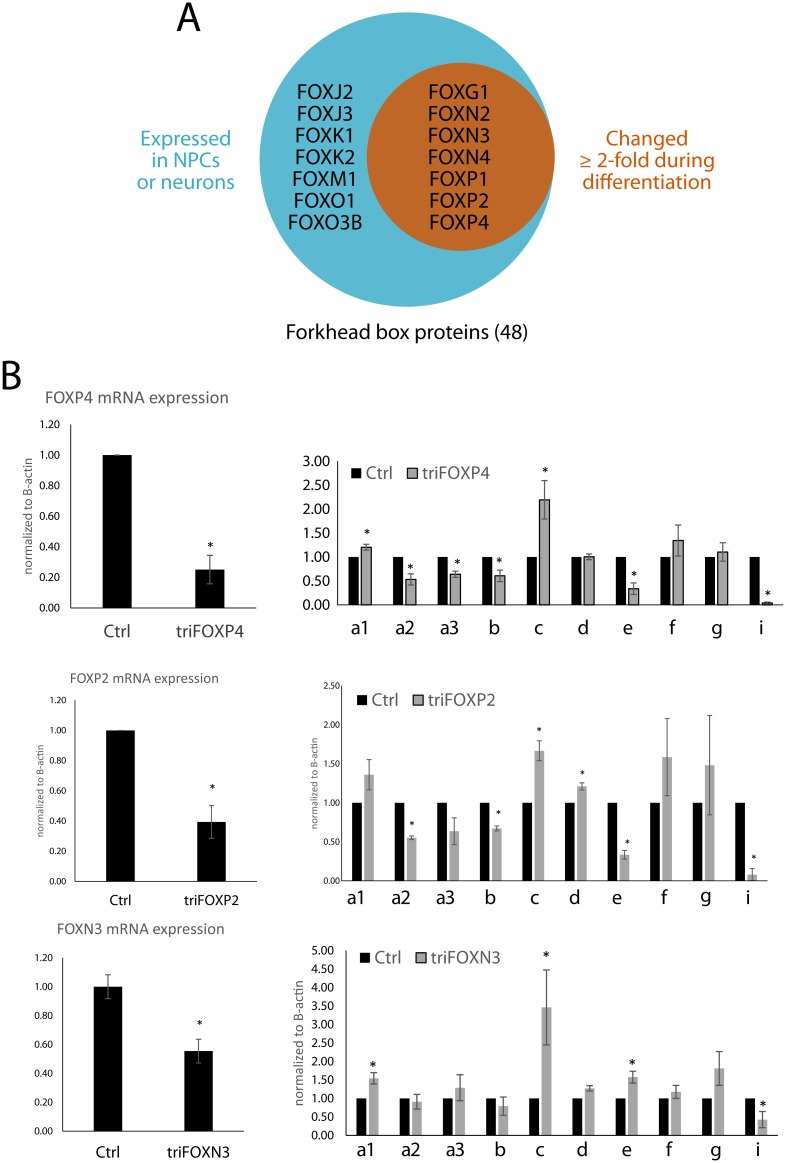
A role for FOX proteins in regulation of pri-let-7s. **(A)** By filtering for FOX genes that are actively transcribed in our neural cells and differentially expressed between PSC-derived NPCs and Tissue-derived NPCs, we generated a list of candidate proteins that might mediate changes in *let-7a3/b* transcription. **(B)** Knockdown of FOX proteins, FOXP2, FOXP4 and FOXN3 in Tissue-derived NPCs show distinct effects on primary *let-7* transcript levels. Statistics were performed across three independent experiments (two-tailed t-test, p < 0.05).

The forkhead box proteins FOXP2, FOXP1, FOXP4, FOXN2, FOXN3, FOXN4, and FOXG1 showed both high baseline expression and dynamic changes in expression over the course of nervous system development (Fig 6A). FOXP2’s role in brain development is linked closely to its involvement in diseases of speech and language[33, 34]. In the murine developing spinal cord and cortex, Foxp2 and Foxp4 are expressed in neural progenitor cells and increase in abundance during neuronal differentiation[33]. In Foxp4^-/-^ mice, these NPCs fail to exit the progenitor stage and cause major disruptions in the developing neural tube. No function in the nervous system has been ascribed to either FOXN2 or FOXN3, but murine Foxn4 is expressed in the brain and retina, and is necessary for the specification of retinal amacrine cells[35]. Taken together, forkhead box proteins have the molecular components necessary to induce reorganizations of the epigenetic state, and some are expressed at anatomic locations and times that correlate with *let-7* expression.

FOXP2, FOXP4, and FOXN3 are all suppressed over the course of our neurodevelopmental model (Fig 6A). Silencing either FOXP4 or FOXP2 in human neural progenitors appeared to inhibit expression of the *let-7a3/b*, *let-7e* and *let-7i* loci, while having nearly the opposite effect on the *let-7a1/d/f1* and *let7c* loci (Fig 6B). On the other hand, silencing FOXN3 did not affect *let-7a3b*, but did induce *let-7c* and *let-7e* (Fig 6B). The fact that the polycistronic let-7 pri-miRNAs appeared to be regulated in concert as a result of these manipulations is further evidence of the co-regulatory mechanisms used during cell fate decision-making. Together, these data demonstrate that proper annotation of *let7* loci can facilitate prediction of regulatory elements that are bound by transcription factors with the ability to regulate *let-7* transcription.

## Discussion

Together, these analyses define contexts in which particular *let-7* polycistrons are transcriptionally regulated, and identify TFs that play roles in this dynamism. This study is not the first to identify transcriptional mechanisms for *let-7* family members, but previous studies from lower organisms did not take advantage of genome-wide analyses to systematically define regulatory modules or transcription factors that regulate them. The fact that *let-7* miRNAs can be dynamically regulated at the transcriptional level has only recently been appreciated, but the relative contribution of this regulation relative to levels of mature *let-7*s remains undefined. This is potentially an important issue to resolve as recent evidence suggests that not all *let-7* miRNAs are processed by the same machinery[36], and therefore, the level of mature *let-7* might not simply be DICER dependent.

These issues bring to light an interesting question, why have mammals evolved to have so many *let-7* isoforms in their genomes, and why do so in polycistronic fashion. Because all the *let-7* family members have the same seed sequence, it seems redundant to express so many. Even in the early neural lineage where mature *let-7*s are scarce, some of the *let-7* polycistrons are not transcribed, whereas others appear to be constitutively expressed. While we can only speculate, it is possible that both dynamic and constitutive *let-7* transcription is a function of feed-back activity of *let-7*-target interactions. It is worth pointing out that some *let-7* targets also regulate *let-7* maturation, such as LIN28A, LIN28B and LIN41. Furthermore, it has been proposed that some *let-7* target RNAs can act as ceRNA or sponges of mature *let-7* to regulate their activity[37]. In addition, some of the TFs shown here and elsewhere to regulate *let-7* transcription (e.g. N-MYC) are also *let-7* target genes[27, 38, 39]. Perhaps, the constitutive transcription and maturation of small amounts of *let-7* serves as something of a rheostat of developmental timing that is tuned as cells become more specified, leading to changes in *let-7* targeted TFs that can then in turn regulate *let-7* transcription, leading to even more mature *let-7* through an additional feed-forward mechanism.

In *C*. *elegans*, where *let-7*s were first discovered, there is evidence for both transcriptional and maturation control despite the fact that all *let-7* is transcribed from a single locus. In fact, there are two distinct transcriptional start sites and these are distinctly regulated by both cis and trans mechanisms. There is further evidence that *let-7*s play a more general role in miRNA biogenesis through an interaction with Argonaute[40, 41]. Therefore, sophisticated mechanisms for *let-7* regulation have been preserved and expanded across evolution, perhaps pointing to their critical roles in both developmental timing and tumorigenesis. These issues are highly relevant to the study of cancer, where *let-7* targets are strongly induced, consistent with a loss of mature *let-7*. It is possible that transcriptional induction of *let-7* family members could be a strategy to drive a cascade of re-expression of *let-7* in cancerous tissues, akin to the process which appears to happen during early human development.

## Materials and Methods

### Cell culture

Pluripotent stem cell culture and differentiation into NPCs and neurons was performed as previously described^6^. Briefly, PSCs were induced to differentiate along the neuroepithelial lineage by treatment with dual inhibitors of the SMAD signaling pathway, SB431542 (Sigma, 5 μM) and LDN193189 (Sigma, 50 nM). Neuroepithelial rosettes were manually picked and replated onto plates coated with ornithine and laminin. Cells were maintained and expanded in NPC media, containing DMEM/F-12 (Gibco), B27 supplement (Gibco), N2 (Gibco), EGF and bFGF. To induce differentiation, cells were fed with media lacking EGF and bFGF for 3 weeks. Tissue-derived NPCs were cultured and differentiated with the same reagents^6^.

### siRNA transfection

Gene knockdowns were performed by transfecting cells with double stranded 27mer RNAs (OriGene) using the lipofectamine RNAiMAX reagent (Thermo Fisher) according to the protocol provided.

### Measurements of gene expression by RT-PCR

Cells were lysed in Trizol lysis reagent (Thermo Fisher), and total RNA was purified from lysates using the QIAgen miRNeasy kit. cDNA was made by reverse transcription from mRNAs with the SuperScript III First Strand Synthesis system (Thermo Fisher), or from miRNAs with the miScript II RT Kit (Qiagen). Realtime PCR was performed on a Roche Lightcycler 480 instrument. For mRNA-derived cDNA, Roche 480 SYBR green I was used. For miRNA-derived cDNA, miScript SYBR (Qiagen) was used. To calculate relative amounts of transcripts, the Real-time data were calculated based on expression levels of housekeeping genes (GAPDH for mRNAs; U6 for miRNAs). miR-15 was included as a control in RT-PCR experiments as this has been proposed to be constitutively expressed in a variety of settings.

### Epigenome characterization and candidate TF prediction

*Summary* ENCODE and Roadmap gene expression data, ChIP-Seq mapping data, and ChromHMM chromatin state prediction were accessed and visualized using the UCSC genome browser and the WashU Epigenome Browser^43,44^. These tools were also used to import and visualize Chromatin-associated RNA-seq reads from Patterson *et al*. and miRNA gene transcripts in cells lacking DGCR8 from Chang *et al*^6,25^.Transcription factor binding site predictions were performed with the ORCA Toolkit web server, and with the MEME suite of motif analysis applications^45,46^.

### Expanded method

The ENCODE and Epigenetic Roadmap datasets, with accession numbers listed in Tables 1 and 2, contain mapped reads from various gene expression and ChIP-sequencing experiments performed on a panel of cell lines and cell types isolated from primary human tissue. These datasets were accessed and imported using the These datasets were accessed and imported using the UCSC genome browser and the WashU Epigenome Browser to co-register and visualize enrichment of epigenetic characteristics across the genome. In figures utilizing Roadmap expression data, track intensity is a logarithmic graph of p-value signal.

**Table 1:** Data from Roadmap project in GEO database.

# GEO Accession	Sample Name	Experiment
GSM772769	brain, angular gyrus	H3K4me3
GSM772770	brain, angular gyrus	H3K4me1
GSM772779	brain, angular gyrus	H3K9me3
GSM772959	brain, angular gyrus	H3K4me3
GSM772960	brain, angular gyrus	H3K9me3
GSM772962	brain, angular gyrus	H3K4me1
GSM772983	brain, angular gyrus	H3K27me3
GSM773016	brain, angular gyrus	H3K27ac
GSM1112807	brain, angular gyrus	H3K27ac
GSM669915	brain, anterior caudate	H3K27me3
GSM669970	brain, anterior caudate	H3K4me1
GSM669994	brain, anterior caudate	H3K9me3
GSM670031	brain, anterior caudate	H3K4me3
GSM772827	brain, anterior caudate	H3K27me3
GSM772829	brain, anterior caudate	H3K4me3
GSM772830	brain, anterior caudate	H3K4me1
GSM772831	brain, anterior caudate	H3K9me3
GSM772832	brain, anterior caudate	H3K27ac
GSM1112811	brain, anterior caudate	H3K27ac
GSM669905	brain, cingulate gyrus	H3K4me3
GSM669923	brain, cingulate gyrus	H3K9me3
GSM670033	brain, cingulate gyrus	H3K4me1
GSM772989	brain, cingulate gyrus	H3K27me3
GSM773007	brain, cingulate gyrus	H3K4me1
GSM773008	brain, cingulate gyrus	H3K4me3
GSM773011	brain, cingulate gyrus	H3K27ac
GSM916023	brain, cingulate gyrus	H3K9me3
GSM1112813	brain, cingulate gyrus	H3K27ac
GSM669624	brain, dorsal neocortex, fetal week15 U	H3K4me3
GSM669625	brain, dorsal neocortex, fetal week15 U	H3K27me3
GSM878650	brain, fetal day101 M	DNase hypersensitivity
GSM878651	brain, fetal day104 M	DNase hypersensitivity
GSM1027328	brain, fetal day105 M	DNase hypersensitivity
GSM878652	brain, fetal day109 F	DNase hypersensitivity
GSM665804	brain, fetal day112 U	DNase hypersensitivity
GSM595920	brain, fetal day117 F	DNase hypersensitivity
GSM706850	brain, fetal day120 U	H3K4me1
GSM916054	brain, fetal day120 U	H3K9me3
GSM916061	brain, fetal day120 U	H3K27me3
GSM530651	brain, fetal day122 M	DNase hypersensitivity
GSM595913	brain, fetal day122 M	DNase hypersensitivity
GSM621393	brain, fetal day122 M	H3K27me3
GSM621427	brain, fetal day122 M	H3K9me3
GSM621457	brain, fetal day122 M	H3K4me3
GSM665819	brain, fetal day142 F	DNase hypersensitivity
GSM595922	brain, fetal day85 F	DNase hypersensitivity
GSM595923	brain, fetal day85 F	DNase hypersensitivity
GSM595926	brain, fetal day96 F	DNase hypersensitivity
GSM595928	brain, fetal day96 F	DNase hypersensitivity
GSM806934	brain, fetal week17 F	H3K4me1
GSM806935	brain, fetal week17 F	H3K4me3
GSM806936	brain, fetal week17 F	H3K9me3
GSM806937	brain, fetal week17 F	H3K27me3
GSM806942	brain, fetal week17 F	H3K4me1
GSM806943	brain, fetal week17 F	H3K4me3
GSM806944	brain, fetal week17 F	H3K9me3
GSM806945	brain, fetal week17 F	H3K27me3
GSM706999	brain, germinal matrix, fetal week20 M	H3K4me3
GSM707000	brain, germinal matrix, fetal week20 M	H3K9me3
GSM707001	brain, germinal matrix, fetal week20 M	H3K27me3
GSM806939	brain, germinal matrix, fetal week20 M	H3K4me1
GSM806940	brain, germinal matrix, fetal week20 M	H3K4me3
GSM806941	brain, germinal matrix, fetal week20 M	H3K9me3
GSM817226	brain, germinal matrix, fetal week20 M	H3K27me3
GSM817228	brain, germinal matrix, fetal week20 M	H3K4me1
GSM669913	brain, hippocampus middle	H3K27me3
GSM669962	brain, hippocampus middle	H3K4me1
GSM670001	brain, hippocampus middle	H3K9me3
GSM670022	brain, hippocampus middle	H3K4me3
GSM773017	brain, hippocampus middle	H3K9me3
GSM773020	brain, hippocampus middle	H3K27ac
GSM773021	brain, hippocampus middle	H3K4me1
GSM773022	brain, hippocampus middle	H3K4me3
GSM916034	brain, hippocampus middle	H3K9me3
GSM916035	brain, hippocampus middle	H3K27ac
GSM916038	brain, hippocampus middle	H3K27me3
GSM916039	brain, hippocampus middle	H3K4me1
GSM916040	brain, hippocampus middle	H3K4me3
GSM1112791	brain, hippocampus middle	H3K27ac
GSM1112800	brain, hippocampus middle	H3K27me3
GSM669992	brain, inferior temporal lobe	H3K4me3
GSM670005	brain, inferior temporal lobe	H3K9me3
GSM670036	brain, inferior temporal lobe	H3K4me1
GSM772772	brain, inferior temporal lobe	H3K27me3
GSM772992	brain, inferior temporal lobe	H3K4me1
GSM772993	brain, inferior temporal lobe	H3K27me3
GSM772994	brain, inferior temporal lobe	H3K9me3
GSM772995	brain, inferior temporal lobe	H3K27ac
GSM772996	brain, inferior temporal lobe	H3K4me3
GSM1112812	brain, inferior temporal lobe	H3K27ac
GSM669965	brain, mid frontal, Brodmann area 9/46, dorsolateral prefrontal cortex	H3K9me3
GSM670015	brain, mid frontal, Brodmann area 9/46, dorsolateral prefrontal cortex	H3K4me1
GSM670016	brain, mid frontal, Brodmann area 9/46, dorsolateral prefrontal cortex	H3K4me3
GSM772833	brain, mid frontal, Brodmann area 9/46, dorsolateral prefrontal cortex	H3K27me3
GSM772834	brain, mid frontal, Brodmann area 9/46, dorsolateral prefrontal cortex	H3K9me3
GSM773012	brain, mid frontal, Brodmann area 9/46, dorsolateral prefrontal cortex	H3K4me3
GSM773014	brain, mid frontal, Brodmann area 9/46, dorsolateral prefrontal cortex	H3K4me1
GSM773015	brain, mid frontal, Brodmann area 9/46, dorsolateral prefrontal cortex	H3K27ac
GSM1112810	brain, mid frontal, Brodmann area 9/46, dorsolateral prefrontal cortex	H3K27ac
GSM669941	brain, substantia nigra	H3K4me1
GSM669953	brain, substantia nigra	H3K27me3
GSM669973	brain, substantia nigra	H3K9me3
GSM670038	brain, substantia nigra	H3K4me3
GSM772897	brain, substantia nigra	H3K9me3
GSM772898	brain, substantia nigra	H3K4me1
GSM772901	brain, substantia nigra	H3K4me3
GSM772937	brain, substantia nigra	H3K27me3
GSM997258	brain, substantia nigra	H3K27ac
GSM1112778	brain, substantia nigra	H3K27ac
GSM537625	ES-I3 cell line	H3K9me3
GSM537626	ES-I3 cell line	H3K4me3
GSM537627	ES-I3 cell line	H3K27me3
GSM537648	ES-I3 cell line	H3K27me3
GSM537664	ES-I3 cell line	H3K9me3
GSM537665	ES-I3 cell line	H3K4me3
GSM537668	ES-I3 cell line	H3K4me1
GSM772789	ES-I3 cell line	H3K4me1
GSM537623	ES-WA7 cell line	H3K4me3
GSM537639	ES-WA7 cell line	H3K9me3
GSM537641	ES-WA7 cell line	H3K4me1
GSM537657	ES-WA7 cell line	H3K27me3
GSM409307	H1 cell line	H3K4me1
GSM409308	H1 cell line	H3K4me3
GSM410808	H1 cell line	H3K4me3
GSM428291	H1 cell line	H3K9me3
GSM428295	H1 cell line	H3K27me3
GSM432392	H1 cell line	H3K4me3
GSM433167	H1 cell line	H3K27me3
GSM433170	H1 cell line	H3K4me3
GSM433174	H1 cell line	H3K9me3
GSM433177	H1 cell line	H3K4me1
GSM434762	H1 cell line	H3K4me1
GSM434776	H1 cell line	H3K27me3
GSM450266	H1 cell line	H3K9me3
GSM466732	H1 cell line	H3K27ac
GSM466734	H1 cell line	H3K27me3
GSM466739	H1 cell line	H3K4me1
GSM469971	H1 cell line	H3K4me3
GSM537679	H1 cell line	H3K4me1
GSM537680	H1 cell line	H3K4me3
GSM537681	H1 cell line	H3K4me3
GSM537683	H1 cell line	H3K27me3
GSM605308	H1 cell line	H3K27me3
GSM605312	H1 cell line	H3K4me1
GSM605315	H1 cell line	H3K4me3
GSM605325	H1 cell line	H3K9me3
GSM605327	H1 cell line	H3K9me3
GSM605328	H1 cell line	H3K9me3
GSM663427	H1 cell line	H3K27ac
GSM818057	H1 cell line	H3K9me3
GSM878616	H1 cell line	DNase hypersensitivity
GSM878621	H1 cell line	DNase hypersensitivity
GSM1185386	H1 cell line	H3K27me3
GSM753429	H1 derived neuronal progenitor cultured cells	H3K27ac
GSM767343	H1 derived neuronal progenitor cultured cells	H3K27ac
GSM767350	H1 derived neuronal progenitor cultured cells	H3K4me3
GSM767351	H1 derived neuronal progenitor cultured cells	H3K4me3
GSM818031	H1 derived neuronal progenitor cultured cells	H3K27ac
GSM818032	H1 derived neuronal progenitor cultured cells	H3K27me3
GSM818033	H1 derived neuronal progenitor cultured cells	H3K27me3
GSM818039	H1 derived neuronal progenitor cultured cells	H3K4me1
GSM818040	H1 derived neuronal progenitor cultured cells	H3K4me1
GSM818043	H1 derived neuronal progenitor cultured cells	H3K4me3
GSM818055	H1 derived neuronal progenitor cultured cells	H3K9me3
GSM818056	H1 derived neuronal progenitor cultured cells	H3K9me3
GSM878615	H1 derived neuronal progenitor cultured cells	DNase hypersensitivity
GSM896162	H1 derived neuronal progenitor cultured cells	H3K27ac
GSM896165	H1 derived neuronal progenitor cultured cells	H3K27me3
GSM906379	H1 derived neuronal progenitor cultured cells	DNase hypersensitivity
GSM956008	H1 derived neuronal progenitor cultured cells	H3K27ac
GSM956010	H1 derived neuronal progenitor cultured cells	H3K27me3
GSM1013146	H1 derived neuronal progenitor cultured cells	H3K4me1
GSM1013151	H1 derived neuronal progenitor cultured cells	H3K4me3
GSM1013158	H1 derived neuronal progenitor cultured cells	H3K9me3
GSM605307	H9 cell line	H3K27ac
GSM605316	H9 cell line	H3K4me3
GSM616128	H9 cell line	H3K4me3
GSM665037	H9 cell line	H3K27ac
GSM667622	H9 cell line	H3K27me3
GSM667626	H9 cell line	H3K4me1
GSM667631	H9 cell line	H3K9me3
GSM667632	H9 cell line	H3K9me3
GSM667633	H9 cell line	H3K9me3
GSM706066	H9 cell line	H3K27me3
GSM706071	H9 cell line	H3K4me1
GSM878612	H9 cell line	DNase hypersensitivity
GSM878613	H9 cell line	DNase hypersensitivity
GSM772738	H9 derived neuron cultured cells	H3K9me3
GSM772776	H9 derived neuron cultured cells	H3K4me3
GSM772785	H9 derived neuron cultured cells	H3K4me1
GSM772787	H9 derived neuron cultured cells	H3K27me3
GSM772736	H9 derived neuronal progenitor cultured cells	H3K4me3
GSM772801	H9 derived neuronal progenitor cultured cells	H3K27me3
GSM772808	H9 derived neuronal progenitor cultured cells	H3K4me1
GSM772810	H9 derived neuronal progenitor cultured cells	H3K9me3
GSM669936	HUES48 cell line	H3K4me3
GSM669942	HUES48 cell line	H3K27me3
GSM669954	HUES48 cell line	H3K4me1
GSM772766	HUES48 cell line	H3K27me3
GSM772780	HUES48 cell line	H3K9me3
GSM772793	HUES48 cell line	H3K4me1
GSM772797	HUES48 cell line	H3K4me3
GSM772799	HUES48 cell line	H3K9me3
GSM997250	HUES48 cell line	H3K27ac
GSM669885	HUES6 cell line	H3K4me1
GSM669886	HUES6 cell line	H3K9me3
GSM669887	HUES6 cell line	H3K27me3
GSM669889	HUES6 cell line	H3K4me3
GSM669891	HUES6 cell line	H3K4me1
GSM669893	HUES6 cell line	H3K4me3
GSM669894	HUES6 cell line	H3K9me3
GSM669897	HUES6 cell line	H3K27me3
GSM1112774	HUES6 cell line	H3K27ac
GSM1112776	HUES6 cell line	H3K27ac
GSM669928	HUES64 cell line	H3K9me3
GSM669966	HUES64 cell line	H3K4me1
GSM669967	HUES64 cell line	H3K4me3
GSM669974	HUES64 cell line	H3K27me3
GSM772750	HUES64 cell line	H3K27me3
GSM772752	HUES64 cell line	H3K4me3
GSM772756	HUES64 cell line	H3K9me3
GSM772800	HUES64 cell line	H3K4me1
GSM772856	HUES64 cell line	H3K9me3
GSM772971	HUES64 cell line	H3K4me1
GSM772977	HUES64 cell line	H3K27me3
GSM772978	HUES64 cell line	H3K4me3
GSM773002	HUES64 cell line	H3K27me3
GSM997249	HUES64 cell line	H3K27ac
GSM1112775	HUES64 cell line	H3K27ac
GSM468792	IMR90 cell line	DNase hypersensitivity
GSM468801	IMR90 cell line	DNase hypersensitivity
GSM469966	IMR90 cell line	H3K27ac
GSM469967	IMR90 cell line	H3K27ac
GSM469968	IMR90 cell line	H3K27me3
GSM469970	IMR90 cell line	H3K4me3
GSM469974	IMR90 cell line	H3K9me3
GSM521887	IMR90 cell line	H3K27ac
GSM521889	IMR90 cell line	H3K27me3
GSM521895	IMR90 cell line	H3K4me1
GSM521897	IMR90 cell line	H3K4me1
GSM521898	IMR90 cell line	H3K4me1
GSM521901	IMR90 cell line	H3K4me3
GSM521913	IMR90 cell line	H3K9me3
GSM521914	IMR90 cell line	H3K9me3
GSM530665	IMR90 cell line	DNase hypersensitivity
GSM530666	IMR90 cell line	DNase hypersensitivity
GSM665801	iPS DF 19.11 cell line	DNase hypersensitivity
GSM706065	iPS DF 19.11 cell line	H3K27ac
GSM706067	iPS DF 19.11 cell line	H3K27me3
GSM706072	iPS DF 19.11 cell line	H3K4me1
GSM706074	iPS DF 19.11 cell line	H3K4me3
GSM706079	iPS DF 19.11 cell line	H3K9me3
GSM752965	iPS DF 19.11 cell line	H3K27ac
GSM752966	iPS DF 19.11 cell line	H3K27ac
GSM752970	iPS DF 19.11 cell line	H3K27me3
GSM752979	iPS DF 19.11 cell line	H3K4me1
GSM752984	iPS DF 19.11 cell line	H3K4me3
GSM752988	iPS DF 19.11 cell line	H3K9me3
GSM665800	iPS DF 19.7 cell line	DNase hypersensitivity
GSM665803	iPS DF 4.7 cell line	DNase hypersensitivity
GSM665802	iPS DF 6.9 cell line	DNase hypersensitivity
GSM706068	iPS DF 6.9 cell line	H3K27me3
GSM706073	iPS DF 6.9 cell line	H3K4me1
GSM706075	iPS DF 6.9 cell line	H3K4me3
GSM706080	iPS DF 6.9 cell line	H3K9me3
GSM752967	iPS DF 6.9 cell line	H3K27ac
GSM752971	iPS DF 6.9 cell line	H3K27me3
GSM752980	iPS DF 6.9 cell line	H3K4me1
GSM752985	iPS DF 6.9 cell line	H3K4me3
GSM752989	iPS DF 6.9 cell line	H3K9me3
GSM537694	iPS-11a cell line	H3K4me3
GSM537687	iPS-15b cell line	H3K4me3
GSM537691	iPS-15b cell line	H3K9me3
GSM621433	iPS-15b cell line	H3K27me3
GSM772767	iPS-15b cell line	H3K4me1
GSM772935	iPS-18a cell line	H3K27me3
GSM773023	iPS-18a cell line	H3K9me3
GSM773028	iPS-18a cell line	H3K4me1
GSM773029	iPS-18a cell line	H3K4me3
GSM773033	iPS-18a cell line	H3K27ac
GSM537671	iPS-18c cell line	H3K4me3
GSM537674	iPS-18c cell line	H3K27me3
GSM537676	iPS-18c cell line	H3K4me3
GSM537678	iPS-18c cell line	H3K9me3
GSM537688	iPS-20b cell line	H3K9me3
GSM537700	iPS-20b cell line	H3K27me3
GSM621416	iPS-20b cell line	H3K27me3
GSM621423	iPS-20b cell line	H3K4me3
GSM772804	iPS-20b cell line	H3K4me1
GSM772842	iPS-20b cell line	H3K9me3
GSM772844	iPS-20b cell line	H3K4me3
GSM772845	iPS-20b cell line	H3K4me1
GSM772847	iPS-20b cell line	H3K27me3
GSM772848	iPS-20b cell line	H3K27ac
GSM707003	neurosphere cultured cells, cortex derived	H3K4me1
GSM707004	neurosphere cultured cells, cortex derived	H3K4me3
GSM707005	neurosphere cultured cells, cortex derived	H3K9me3
GSM707006	neurosphere cultured cells, cortex derived	H3K27me3
GSM817230	neurosphere cultured cells, cortex derived	H3K4me1
GSM817231	neurosphere cultured cells, cortex derived	H3K9me3
GSM941713	neurosphere cultured cells, cortex derived	H3K27me3
GSM707008	neurosphere cultured cells, ganglionic eminence derived	H3K4me1
GSM707009	neurosphere cultured cells, ganglionic eminence derived	H3K4me3
GSM707010	neurosphere cultured cells, ganglionic eminence derived	H3K9me3
GSM707011	neurosphere cultured cells, ganglionic eminence derived	H3K27me3
GSM817232	neurosphere cultured cells, ganglionic eminence derived	H3K4me1
GSM817233	neurosphere cultured cells, ganglionic eminence derived	H3K9me3
GSM941715	neurosphere cultured cells, ganglionic eminence derived	H3K27me3
GSM1127062	neurosphere cultured cells, ganglionic eminence derived	H3K4me3
GSM1127066	neurosphere cultured cells, ganglionic eminence derived	H3K4me1
GSM1127078	neurosphere cultured cells, ganglionic eminence derived	H3K27me3
GSM1127079	neurosphere cultured cells, ganglionic eminence derived	H3K9me3
GSM1127083	neurosphere cultured cells, ganglionic eminence derived	H3K27ac
GSM941748	UCSF-4 cell line	H3K4me1
GSM941749	UCSF-4 cell line	H3K4me3
GSM941750	UCSF-4 cell line	H3K9me3
GSM941751	UCSF-4 cell line	H3K27me3
GSM1127063	UCSF-4 cell line	H3K4me3
GSM1127080	UCSF-4 cell line	H3K4me1
GSM1127081	UCSF-4 cell line	H3K9me3
GSM1127084	UCSF-4 cell line	H3K27me3

**Table 2:** Data from ENCODE project in GEO database (or DCC Accession #).

GEO Accession #	Sample Cell Line	ChIP Antibody	DCC Accession
GSM749677	BJ	CTCF	wgEncodeEH000403
	BJ	H3K27me3	wgEncodeEH000424
GSM945207	BJ	H3K36me3	wgEncodeEH000443
GSM945178	BJ	H3K4me3	wgEncodeEH000416
GSM822281	Fibrobl	CTCF	wgEncodeEH001127
GSM822303	Gliobla	CTCF	wgEncodeEH001135
GSM822302	Gliobla	Pol2	wgEncodeEH001136
	H1-hESC	ATF2	wgEncodeEH002316
GSM803512	H1-hESC	ATF3	wgEncodeEH001566
	H1-hESC	Bach1	wgEncodeEH002842
GSM803396	H1-hESC	BCL11A	wgEncodeEH001527
GSM803476	H1-hESC	BCL11A	wgEncodeEH001625
GSM935517	H1-hESC	BRCA1	wgEncodeEH002801
	H1-hESC	CEBPB	wgEncodeEH002825
GSM1003444	H1-hESC	CHD1	wgEncodeEH002095
GSM935296	H1-hESC	CHD1	wgEncodeEH002826
GSM935297	H1-hESC	CHD2	wgEncodeEH002827
GSM1003473	H1-hESC	CHD7	wgEncodeEH003136
GSM935614	H1-hESC	c-Jun	wgEncodeEH001854
GSM822274	H1-hESC	c-Myc	wgEncodeEH000596
	H1-hESC	c-Myc	wgEncodeEH002795
	H1-hESC	CREB1	wgEncodeEH003229
	H1-hESC	CtBP2	wgEncodeEH001767
GSM733672	H1-hESC	CTCF	wgEncodeEH000085
GSM822297	H1-hESC	CTCF	wgEncodeEH000560
GSM803419	H1-hESC	CTCF	wgEncodeEH001649
GSM1010899	H1-hESC	E2F6	wgEncodeEH003224
GSM803430	H1-hESC	Egr-1	wgEncodeEH001538
GSM1003524	H1-hESC	EZH2	wgEncodeEH003082
GSM803382	H1-hESC	FOSL1	wgEncodeEH001660
GSM803424	H1-hESC	GABP	wgEncodeEH001534
GSM935581	H1-hESC	GTF2F1	wgEncodeEH002843
GSM1003579	H1-hESC	H2A.Z	wgEncodeEH002082
GSM733718	H1-hESC	H3K27ac	wgEncodeEH000997
GSM733748	H1-hESC	H3K27me3	wgEncodeEH000074
GSM733725	H1-hESC	H3K36me3	wgEncodeEH000107
GSM733782	H1-hESC	H3K4me1	wgEncodeEH000106
GSM733670	H1-hESC	H3K4me2	wgEncodeEH000108
GSM733657	H1-hESC	H3K4me3	wgEncodeEH000086
	H1-hESC	H3K79me2	wgEncodeEH002083
GSM733773	H1-hESC	H3K9ac	wgEncodeEH000109
GSM1003585	H1-hESC	H3K9me3	wgEncodeEH002084
GSM733687	H1-hESC	H4K20me1	wgEncodeEH000087
GSM803345	H1-hESC	HDAC2	wgEncodeEH001659
GSM1003472	H1-hESC	HDAC2	wgEncodeEH003137
GSM1003571	H1-hESC	HDAC6	wgEncodeEH003100
	H1-hESC	JARID1A	wgEncodeEH002096
GSM1003479	H1-hESC	JMJD2A	wgEncodeEH003138
GSM803529	H1-hESC	JunD	wgEncodeEH001579
GSM935434	H1-hESC	JunD	wgEncodeEH002023
	H1-hESC	MafK	wgEncodeEH002828
GSM935348	H1-hESC	Max	wgEncodeEH001757
	H1-hESC	Max	wgEncodeEH003225
	H1-hESC	Max	wgEncodeEH003359
	H1-hESC	Mxi1	wgEncodeEH002829
GSM803437	H1-hESC	NANOG	wgEncodeEH001635
GSM935308	H1-hESC	Nrf1	wgEncodeEH001847
GSM803365	H1-hESC	NRSF	wgEncodeEH001498
GSM803542	H1-hESC	p300	wgEncodeEH001574
GSM1003513	H1-hESC	P300	wgEncodeEH003126
GSM1003509	H1-hESC	PHF8	wgEncodeEH003094
GSM1003457	H1-hESC	PLU1	wgEncodeEH003127
GSM822300	H1-hESC	Pol2	wgEncodeEH000563
GSM803366	H1-hESC	Pol2	wgEncodeEH001499
GSM803484	H1-hESC	Pol2-4H8	wgEncodeEH001514
GSM803438	H1-hESC	POU5F1	wgEncodeEH001636
GSM803466	H1-hESC	Rad21	wgEncodeEH001593
	H1-hESC	Rad21	wgEncodeEH001836
	H1-hESC	RBBP5	wgEncodeEH002087
GSM935382	H1-hESC	RFX5	wgEncodeEH001835
GSM803506	H1-hESC	RXRA	wgEncodeEH001560
GSM1003572	H1-hESC	SAP30	wgEncodeEH003101
GSM935289	H1-hESC	SIN3A	wgEncodeEH002854
GSM803428	H1-hESC	Sin3Ak-20	wgEncodeEH001530
GSM1003451	H1-hESC	SIRT6	wgEncodeEH003128
GSM803405	H1-hESC	SIX5	wgEncodeEH001528
GSM803377	H1-hESC	SP1	wgEncodeEH001529
	H1-hESC	SP2	wgEncodeEH002302
GSM1010743	H1-hESC	SP4	wgEncodeEH002317
GSM803425	H1-hESC	SRF	wgEncodeEH001533
	H1-hESC	SUZ12	wgEncodeEH001752
GSM1003573	H1-hESC	SUZ12	wgEncodeEH003102
GSM803450	H1-hESC	TAF1	wgEncodeEH001500
GSM803501	H1-hESC	TAF7	wgEncodeEH001610
GSM935303	H1-hESC	TBP	wgEncodeEH001848
GSM803427	H1-hESC	TCF12	wgEncodeEH001531
GSM1010845	H1-hESC	TEAD4	wgEncodeEH003214
GSM803426	H1-hESC	USF-1	wgEncodeEH001532
GSM935380	H1-hESC	USF2	wgEncodeEH001837
GSM803513	H1-hESC	YY1	wgEncodeEH001567
	H1-hESC	Znf143	wgEncodeEH002802
GSM1003619	H1-hESC	ZNF274	wgEncodeEH003357
GSM1010804	H1-neurons	NRSF	wgEncodeEH003264
	H1-neurons	Pol2-4H8	wgEncodeEH003265
	H1-neurons	TAF1	wgEncodeEH003266
GSM749668	HEK293	CTCF	wgEncodeEH000396
GSM935590	HEK293	ELK4	wgEncodeEH001773
GSM945288	HEK293	H3K4me3	wgEncodeEH000953
GSM935592	HEK293	KAP1	wgEncodeEH001779
GSM935534	HEK293	Pol2	wgEncodeEH000632
GSM782124	HEK293	TCF7L2	wgEncodeEH002022
GSM935336	K562	ARID3A	wgEncodeEH002861
GSM935340	K562	ATF1	wgEncodeEH002865
GSM935391	K562	ATF3	wgEncodeEH000700
GSM803380	K562	ATF3	wgEncodeEH001662
	K562	Bach1	wgEncodeEH002846
GSM803518	K562	BCL3	wgEncodeEH001570
GSM803515	K562	BCLAF1	wgEncodeEH001571
	K562	BDP1	wgEncodeEH000678
	K562	BHLHE40	wgEncodeEH001857
GSM935595	K562	BRF1	wgEncodeEH000679
GSM935490	K562	BRF2	wgEncodeEH000767
GSM935633	K562	Brg1	wgEncodeEH000724
GSM1003574	K562	CBP	wgEncodeEH003103
GSM1003567	K562	CBX2	wgEncodeEH003104
GSM1010732	K562	CBX3	wgEncodeEH002383
	K562	CBX3	wgEncodeEH003105
	K562	CBX8	wgEncodeEH003106
GSM935547	K562	CCNT2	wgEncodeEH001864
GSM1003622	K562	CDP	wgEncodeEH003391
GSM935499	K562	CEBPB	wgEncodeEH001821
	K562	CEBPB	wgEncodeEH002346
GSM1010906	K562	CEBPD	wgEncodeEH003432
	K562	c-Fos	wgEncodeEH000619
	K562	CHD1	wgEncodeEH002088
	K562	CHD2	wgEncodeEH001822
GSM1003510	K562	CHD4	wgEncodeEH003095
GSM1003478	K562	CHD7	wgEncodeEH003139
GSM935411	K562	c-Jun	wgEncodeEH000620
GSM1003609	K562	c-Jun	wgEncodeEH003369
GSM822310	K562	c-Myc	wgEncodeEH000536
GSM935410	K562	c-Myc	wgEncodeEH000621
GSM935516	K562	c-Myc	wgEncodeEH002800
	K562	COREST	wgEncodeEH002814
	K562	COREST	wgEncodeEH002847
	K562	CREB1	wgEncodeEH003230
GSM733719	K562	CTCF	wgEncodeEH000042
GSM749690	K562	CTCF	wgEncodeEH000399
GSM822311	K562	CTCF	wgEncodeEH000535
GSM1010820	K562	CTCF	wgEncodeEH002279
	K562	CTCF	wgEncodeEH002279
GSM935407	K562	CTCF	wgEncodeEH002797
GSM803401	K562	CTCFL	wgEncodeEH001652
GSM935600	K562	E2F4	wgEncodeEH000671
GSM935597	K562	E2F6	wgEncodeEH000676
GSM803469	K562	E2F6	wgEncodeEH001598
	K562	eGFP-BACH1	wgEncodeEH002986
	K562	eGFP-CCNE1	wgEncodeEH002996
	K562	eGFP-CDKN1B	wgEncodeEH002997
	K562	eGFP-ELF1	wgEncodeEH002998
	K562	eGFP-ESR	wgEncodeEH002999
GSM777644	K562	eGFP-FOS	wgEncodeEH001207
GSM777641	K562	eGFP-GATA2	wgEncodeEH001208
GSM777640	K562	eGFP-HDAC8	wgEncodeEH001209
	K562	eGFP-ILF2	wgEncodeEH003000
GSM777638	K562	eGFP-JunB	wgEncodeEH001210
GSM777639	K562	eGFP-JunD	wgEncodeEH001211
	K562	eGFP-MLL5	wgEncodeEH003001
	K562	eGFP-NCOR1	wgEncodeEH003002
	K562	eGFP-NF90	wgEncodeEH003003
GSM777637	K562	eGFP-NR4A1	wgEncodeEH001212
	K562	eGFP-STAT1	wgEncodeEH003004
GSM803414	K562	Egr-1	wgEncodeEH001646
GSM803494	K562	ELF1	wgEncodeEH001619
	K562	ELK1	wgEncodeEH003356
GSM803442	K562	ETS1	wgEncodeEH001580
	K562	EZH2	wgEncodeEH002089
GSM803439	K562	FOSL1	wgEncodeEH001637
GSM803524	K562	GABP	wgEncodeEH001604
GSM1003608	K562	GATA1	wgEncodeEH003368
GSM935540	K562	GATA-1	wgEncodeEH000638
GSM803540	K562	GATA2	wgEncodeEH001576
GSM935373	K562	GATA-2	wgEncodeEH000683
GSM935394	K562	GTF2B	wgEncodeEH000703
GSM935501	K562	GTF2F1	wgEncodeEH001823
GSM733786	K562	H2A.Z	wgEncodeEH001038
GSM733656	K562	H3K27ac	wgEncodeEH000043
GSM733658	K562	H3K27me3	wgEncodeEH000044
GSM945228	K562	H3K27me3	wgEncodeEH000434
GSM788088	K562	H3K27me3B	wgEncodeEH000912
GSM733714	K562	H3K36me3	wgEncodeEH000045
GSM945302	K562	H3K36me3	wgEncodeEH000435
GSM733692	K562	H3K4me1	wgEncodeEH000046
GSM788085	K562	H3K4me1	wgEncodeEH000911
GSM733651	K562	H3K4me2	wgEncodeEH000047
GSM733680	K562	H3K4me3	wgEncodeEH000048
	K562	H3K4me3	wgEncodeEH000400
GSM788087	K562	H3K4me3B	wgEncodeEH000913
GSM733653	K562	H3K79me2	wgEncodeEH001039
GSM733778	K562	H3K9ac	wgEncodeEH000049
GSM788082	K562	H3K9acB	wgEncodeEH000914
GSM733777	K562	H3K9me1	wgEncodeEH000050
GSM733776	K562	H3K9me3	wgEncodeEH001040
GSM733675	K562	H4K20me1	wgEncodeEH000051
	K562	HCFC1	wgEncodeEH003392
GSM1003448	K562	HDAC1	wgEncodeEH002090
GSM803471	K562	HDAC2	wgEncodeEH001622
GSM1003447	K562	HDAC2	wgEncodeEH002091
	K562	HDAC6	wgEncodeEH003093
GSM803385	K562	HEY1	wgEncodeEH001481
	K562	HMGN3	wgEncodeEH001863
GSM935634	K562	Ini1	wgEncodeEH000725
GSM935569	K562	JunD	wgEncodeEH002164
GSM935464	K562	KAP1	wgEncodeEH001764
GSM1003570	K562	LSD1	wgEncodeEH003107
	K562	MafF	wgEncodeEH002804
GSM935311	K562	MafK	wgEncodeEH001844
GSM935539	K562	Max	wgEncodeEH000637
GSM803523	K562	Max	wgEncodeEH001605
GSM935344	K562	Max	wgEncodeEH002869
GSM935337	K562	MAZ	wgEncodeEH002862
GSM803379	K562	MEF2A	wgEncodeEH001663
GSM935497	K562	Mxi1	wgEncodeEH001827
	K562	NCoR	wgEncodeEH003108
GSM935392	K562	NELFe	wgEncodeEH000701
	K562	NF-E2	wgEncodeEH000624
	K562	NF-YA	wgEncodeEH002021
GSM935429	K562	NF-YB	wgEncodeEH002024
GSM1010782	K562	NR2F2	wgEncodeEH002382
GSM935361	K562	Nrf1	wgEncodeEH001796
GSM803440	K562	NRSF	wgEncodeEH001638
GSM1003492	K562	NSD2	wgEncodeEH003140
GSM935494	K562	p300	wgEncodeEH001828
GSM1003583	K562	p300	wgEncodeEH002086
GSM935401	K562	p300	wgEncodeEH002834
GSM1003566	K562	PCAF	wgEncodeEH003109
GSM1003450	K562	PHF8	wgEncodeEH002092
GSM1003586	K562	PLU1	wgEncodeEH002085
GSM1010722	K562	PML	wgEncodeEH002320
GSM822275	K562	Pol2	wgEncodeEH000555
	K562	Pol2	wgEncodeEH000616
GSM935632	K562	Pol2	wgEncodeEH000727
GSM803410	K562	Pol2	wgEncodeEH001633
GSM733643	K562	Pol2(b)	wgEncodeEH000053
GSM935645	K562	Pol2(phosphoS2)	wgEncodeEH001805
	K562	Pol2(phosphoS2)	wgEncodeEH002833
GSM803443	K562	Pol2-4H8	wgEncodeEH001581
GSM935481	K562	Pol3	wgEncodeEH000694
GSM803384	K562	PU.1	wgEncodeEH001482
GSM935319	K562	Rad21	wgEncodeEH000649
GSM803447	K562	Rad21	wgEncodeEH001585
	K562	RBBP5	wgEncodeEH002093
GSM1003507	K562	REST	wgEncodeEH003096
GSM935565	K562	RFX5	wgEncodeEH002033
GSM1003563	K562	RNF2	wgEncodeEH003110
GSM935372	K562	RPC155	wgEncodeEH000680
GSM1003445	K562	SAP30	wgEncodeEH002094
GSM935598	K562	SETDB1	wgEncodeEH000677
GSM1003452	K562	SETDB1	wgEncodeEH003129
GSM803525	K562	Sin3Ak-20	wgEncodeEH001607
	K562	SIRT6	wgEncodeEH000681
GSM1003560	K562	SIRT6	wgEncodeEH003111
GSM803383	K562	SIX5	wgEncodeEH001483
GSM803378	K562	SIX5	wgEncodeEH001664
	K562	SMC3	wgEncodeEH001845
GSM803505	K562	SP1	wgEncodeEH001578
GSM803402	K562	SP2	wgEncodeEH001653
GSM803520	K562	SRF	wgEncodeEH001600
GSM1010877	K562	STAT5A	wgEncodeEH002347
GSM1003545	K562	SUZ12	wgEncodeEH003112
GSM803431	K562	TAF1	wgEncodeEH001582
GSM803407	K562	TAF7	wgEncodeEH001654
	K562	TAL1	wgEncodeEH001824
GSM935574	K562	TBLR1	wgEncodeEH002848
	K562	TBLR1	wgEncodeEH002849
GSM935495	K562	TBP	wgEncodeEH001825
	K562	TEAD4	wgEncodeEH002333
GSM935343	K562	TFIIIC-110	wgEncodeEH000748
GSM803408	K562	THAP1	wgEncodeEH001655
GSM935374	K562	TR4	wgEncodeEH000682
GSM1010849	K562	TRIM28	wgEncodeEH003210
GSM935338	K562	UBF	wgEncodeEH002863
	K562	UBTF	wgEncodeEH002850
GSM803441	K562	USF-1	wgEncodeEH001583
	K562	USF2	wgEncodeEH001797
GSM935425	K562	XRCC4	wgEncodeEH000650
	K562	YY1	wgEncodeEH000684
GSM803446	K562	YY1	wgEncodeEH001584
GSM803470	K562	YY1	wgEncodeEH001623
GSM803504	K562	ZBTB33	wgEncodeEH001569
GSM803473	K562	ZBTB7A	wgEncodeEH001620
	K562	ZC3H11A	wgEncodeEH003380
GSM935568	K562	Znf143	wgEncodeEH002030
	K562	ZNF263	wgEncodeEH000630
GSM935479	K562	ZNF274	wgEncodeEH000696
GSM935503	K562	ZNF274	wgEncodeEH002068
GSM1003621	K562	ZNF384	wgEncodeEH003382
GSM1003611	K562	ZNF-MIZD-CP1	wgEncodeEH003381
GSM733744	NHDF-Ad	CTCF	wgEncodeEH001048
	NHDF-Ad	EZH2	wgEncodeEH002438
GSM1003505	NHDF-Ad	H2A.Z	wgEncodeEH003090
GSM733662	NHDF-Ad	H3K27ac	wgEncodeEH001049
GSM733745	NHDF-Ad	H3K27me3	wgEncodeEH001050
GSM733733	NHDF-Ad	H3K36me3	wgEncodeEH001051
GSM1003526	NHDF-Ad	H3K4me1	wgEncodeEH002429
GSM733753	NHDF-Ad	H3K4me2	wgEncodeEH001052
GSM733650	NHDF-Ad	H3K4me3	wgEncodeEH001053
GSM1003554	NHDF-Ad	H3K79me2	wgEncodeEH002430
GSM733709	NHDF-Ad	H3K9ac	wgEncodeEH001054
GSM1003553	NHDF-Ad	H3K9me3	wgEncodeEH002431
GSM1003486	NHDF-Ad	H4K20me1	wgEncodeEH002417
	WI-38	CTCF	wgEncodeEH001902
GSM945265	WI-38	H3K4me3	wgEncodeEH001914

The genome regions surrounding known *let-7* gene locations were surveyed for the presence of histone modifications and open chromatin in cell types representative of the stages of differentiation from PSCs to NPCs and neurons. We also imported miRNA gene transcripts in cells lacking DGCR8 from Chang *et al*, which are accessible using the NIH SRA database, accession SRP057660. Together, these data allowed us to identify primary *let-7* transcripts, based on their expression in our Chromatin-associated RNA-seq samples and in DGCR8^-/-^ RNA-seq samples, even when they disagreed with RefSeq-annotated *MIRLET7* genes. Hypothesized regulatory regions were assembled by searching 20 kilobases upstream and downstream of each transcript for colocalization of H3K27Ac, H3K4me3, and DNAse sensitivity in samples known to express *let-7* primary transcripts, and H3K27me3 or H3K9me3 in samples without appreciable primary *let-7* transcripts. These regions were frequently annotated as Active TSS, Flanking active TSS, Bivalent/Poised TSS, Enhancer, Genic Enhancer, and Bivalent Enhancer in the ChromHMM chromatin state prediction algorithm performed on Roadmap datasets.

These hypothesized regulatory regions were then queried for transcription factor binding sites, based on known and predicted TF binding sites and motifs. We used both the ORCA Toolkit web server and the MEME suite of motif analysis applications to isolate highly conserved (>80% phastCons score) sub-regions within these hypothesized regulatory regions, and then queried those conserved sub-regions for TF motifs. These motifs were assembled from the JASPAR motif database as well as a small list of manually curated motif sequences. Where possible, experimental ChIP-Seq validated TF binding sites from ENCODE and ROADMAP dataset were used as secondary validation of predicted TF binding sites. Accession numbers for these datasets are also found in Tables 1 and 2.

## Supporting Information

S1 Fig Complete annotation of let-7 miRNA transcripts and regulation in human PSCs and NPCs by Chromatin RNA-seq. Shown are each of the let-7 family member transcripts, including polycistrons. The top of the graphic shows the genomic locus. The middle section are data from the Chromatin RNA-seq described in Fig 1. Below in green are the annotations for let-7 miRNAs described in Cheng et al in the indicated cell types. Below in blue are the annotations according to public genome browsers.
(PDF)

S2 Fig Annotation of epigenetic marks at two let-7 polycistronic loci. Epigenetic marks from the Roadmap Epigenomics project at the dynamic (let-7a3/b) and constitutive (let-7a1/d1/f1) polycistronic loci. At top are the Chromatin-associated RNA-Seq peaks and RefSeq annotations of the primary *let-7* transcripts, and below are the relative intensities of DNAse sensitivity or histone modification ChIP-Seq peaks at those loci.
(PDF)

S3 Fig Complete annotation of let-7 miRNA transcripts and summary of available data on epigenetic marks across various cell types. Shown are the let-7 genomic loci with accompanying epigenetic marks as identified by ChIP-seq data available from the epigenetic roadmap across the indicated cell types. The bottom portion also includes available ChIP-seq data on the indicated transcription factor binding patterns at these same loci.
(PDF)
