# Supplementary figures and images for: Defining Transcriptional Regulatory Mechanisms for Primary let-7 miRNAs
Source: PLoS One. 2017 Jan 4;12(1):e0169237. doi: 10.1371/journal.pone.0169237 (PMC5215532; doi:10.1371/journal.pone.0169237)

**let-7-a2 locus**  
Chr11 q24.1

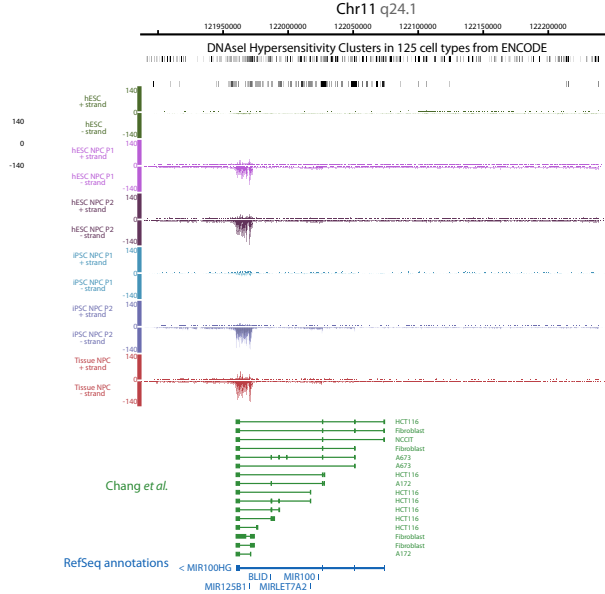

**let-7-c locus**  
Chr21 q21.1

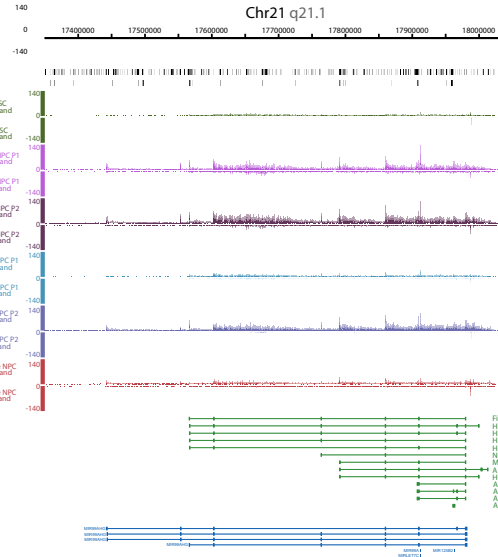

**let-7e- locus**  
Chr19 q13.41

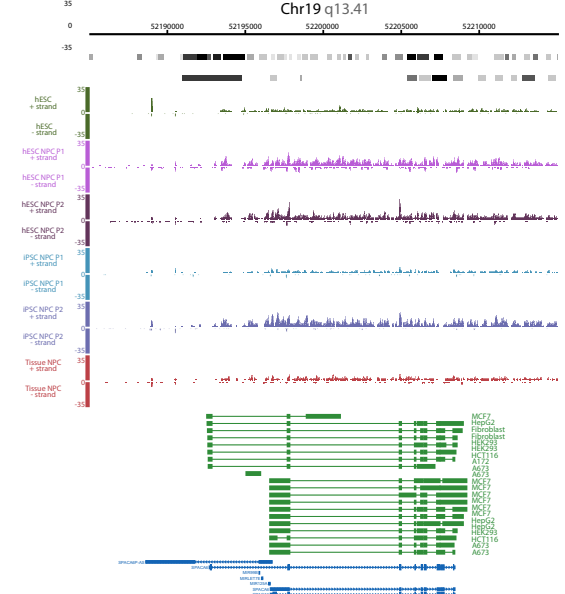

**let-7f2- locus**  
ChrX p11.22

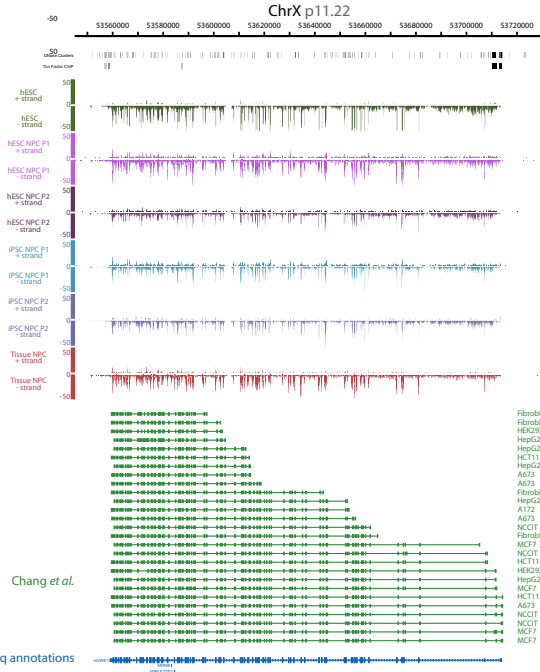

**let-7g- locus**  
Chr3 p21.2-21.1

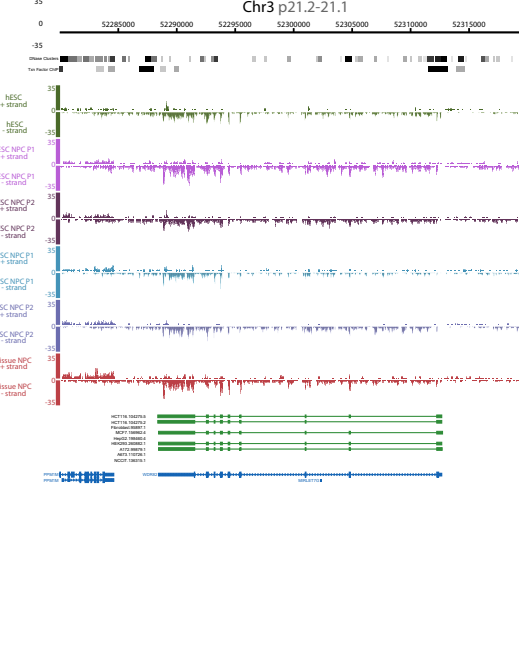

Supplement: S1 Fig — Shown are each of the let-7 family member transcripts, including polycistrons. The top of the graphic shows the genomic locus. The middle section are data from the Chromatin RNA-seq described in Fig 1. Below in green are the annotations for let-7 miRNAs described in Cheng et al in the indicated cell types. Below in blue are the annotations according to public genome browsers. (PDF) [file pone.0169237.s001.pdf]

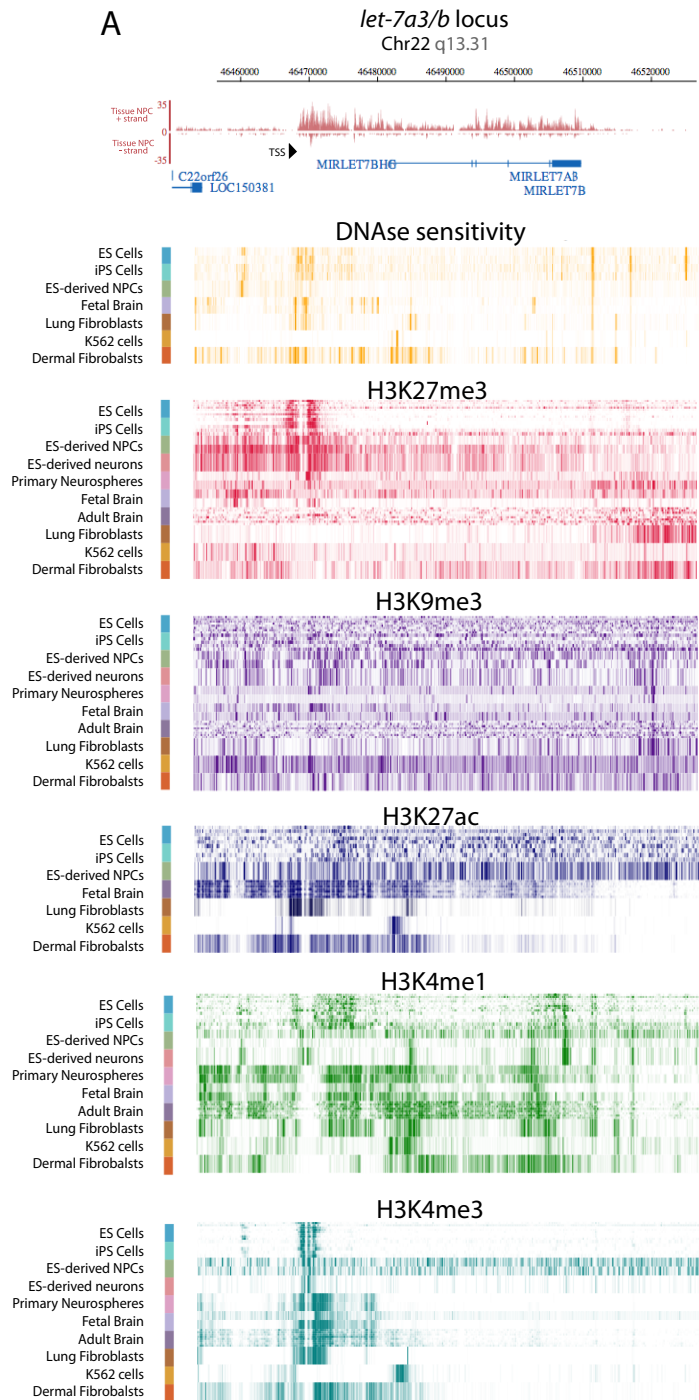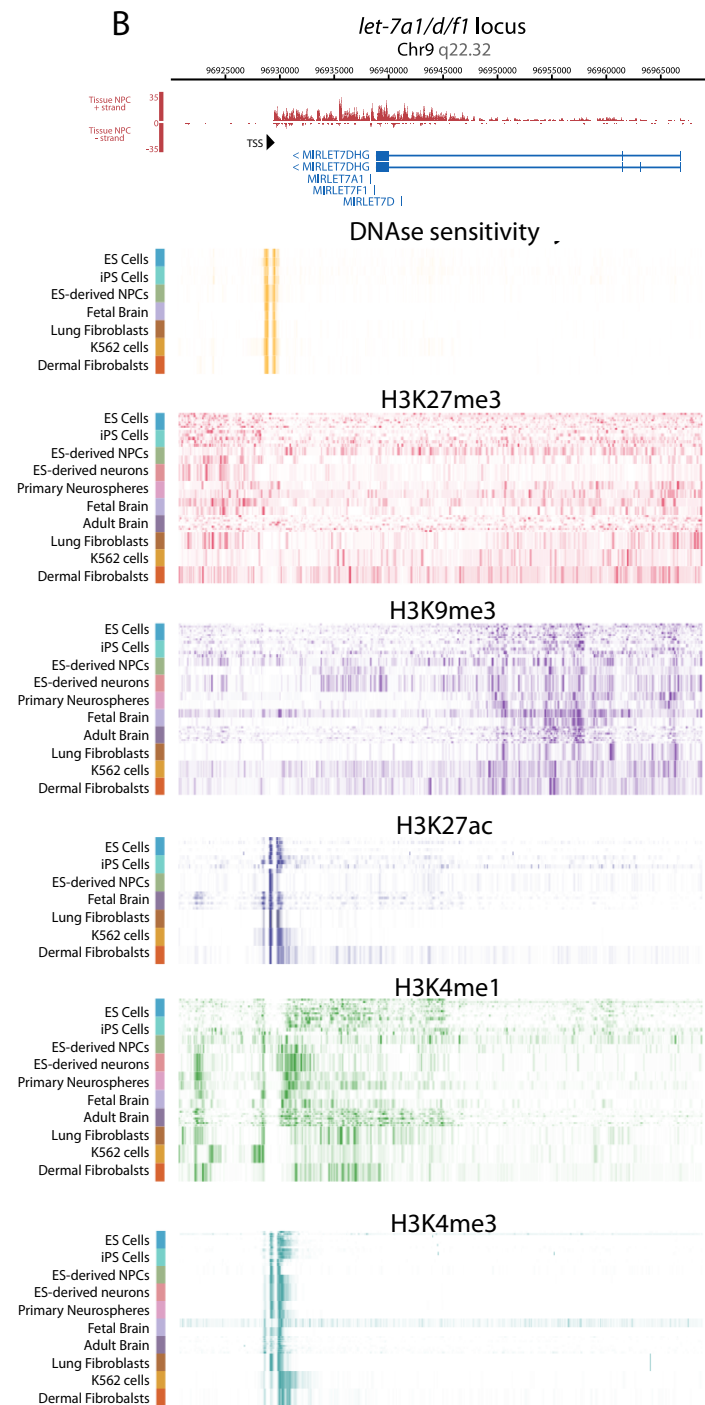

Supplement: S2 Fig — Epigenetic marks from the Roadmap Epigenomics project at the dynamic (let-7a3/b) and constitutive (let-7a1/d1/f1) polycistronic loci. At top are the Chromatin-associated RNA-Seq peaks and RefSeq annotations of the primary let-7 transcripts, and below are the relative intensities of DNAse sensitivity or histone modification ChIP-Seq peaks at those loci. (PDF) [file pone.0169237.s002.pdf]

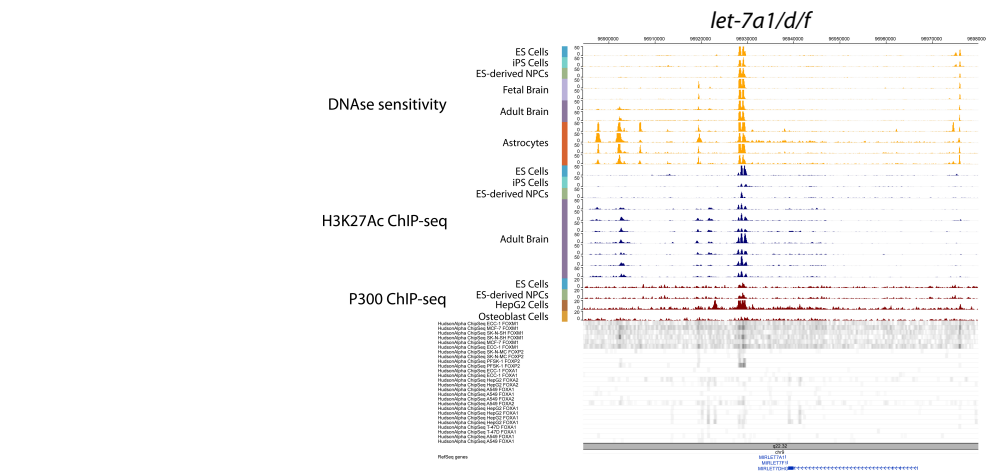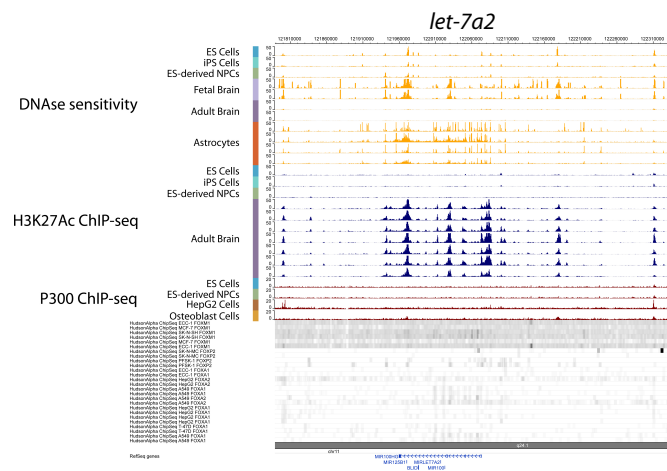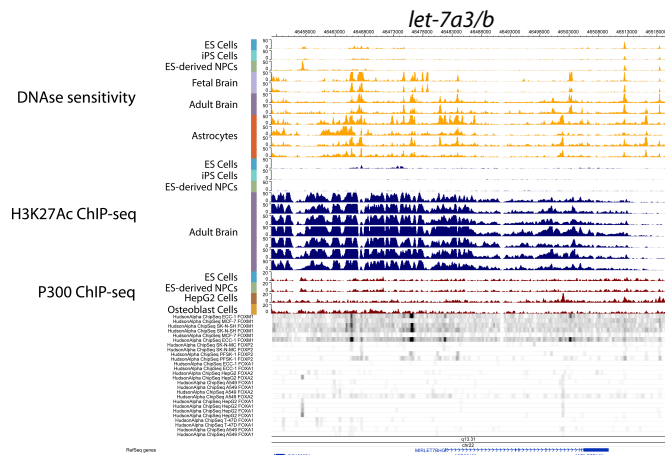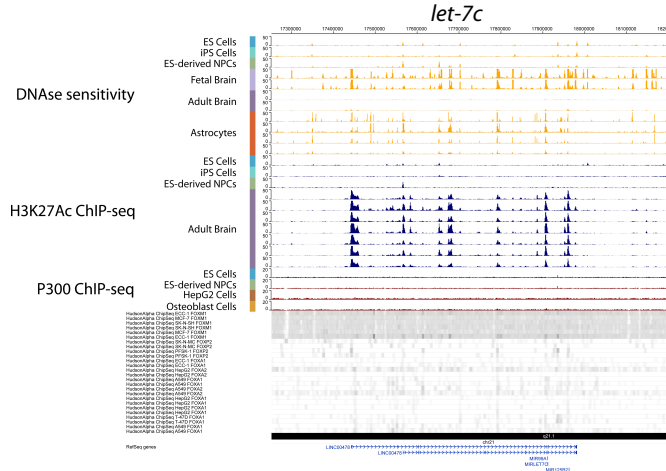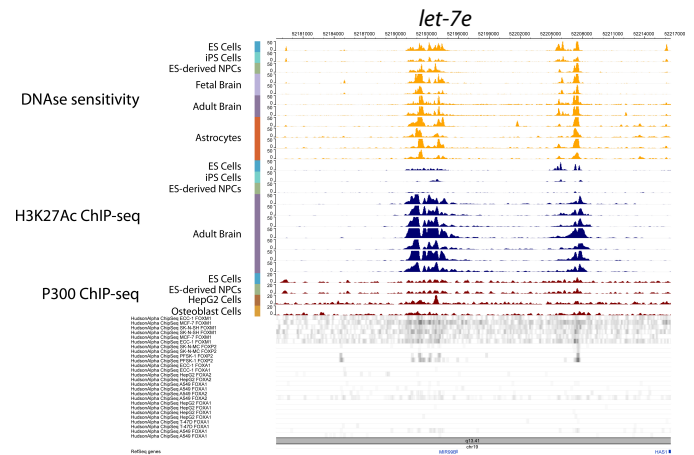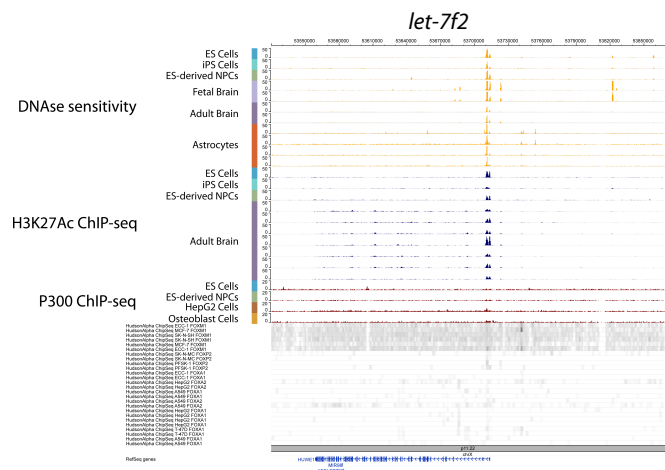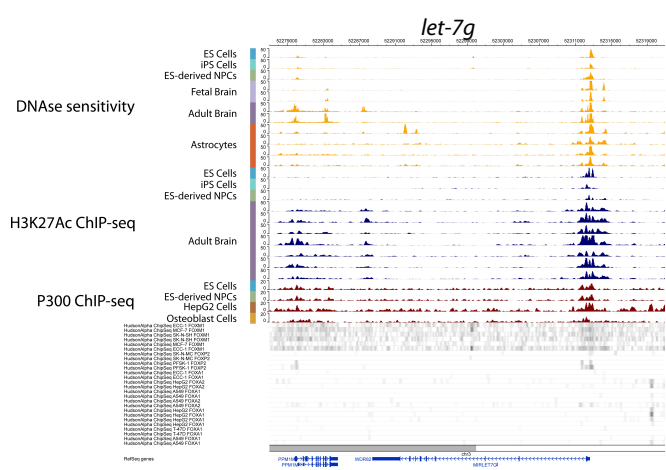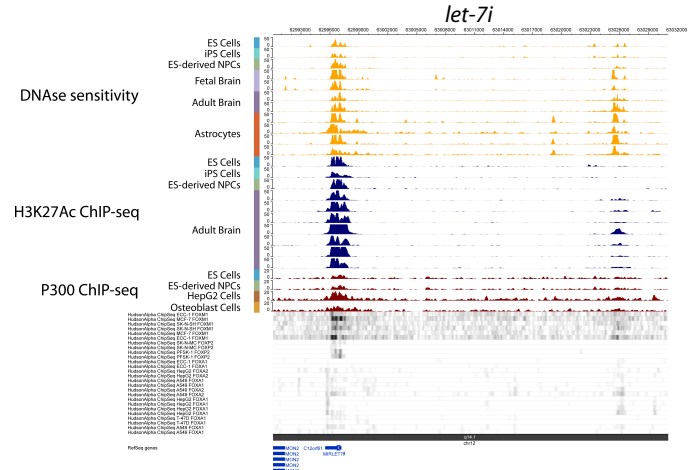

Supplement: S3 Fig — Shown are the let-7 genomic loci with accompanying epigenetic marks as identified by ChIP-seq data available from the epigenetic roadmap across the indicated cell types. The bottom portion also includes available ChIP-seq data on the indicated transcription factor binding patterns at these same loci. (PDF) [file pone.0169237.s003.pdf]
